# Outbreak of porcine epidemic diarrhoea virus (PEDV) in Abruzzi region, central‐Italy

**DOI:** 10.1002/vms3.88

**Published:** 2017-12-18

**Authors:** Federica Pizzurro, Francesca Cito, Guendalina Zaccaria, Massimo Spedicato, Angelo Cerella, Massimiliano Orsini, Mario Forzan, Nicola D'Alterio, Alessio Lorusso, Maurilia Marcacci

**Affiliations:** ^1^ National Reference Center for Whole Genome Sequencing of microbial pathogens‐ Istituto Zooprofilattico Sperimentale dell'Abruzzo e del Molise (IZSAM) Teramo Italy; ^2^ Dipartimento di Scienze Veterinarie Università di Pisa Pisa Italy

**Keywords:** Porcine epidemic diarrhoea virus, Outbreak, Phylogenetic analysis, Spike gene, S‐INDEL

## Abstract

Here we report and characterize a porcine epidemic diarrhea (PED) outbreak which occurred in a swine fattening farm in the province of Teramo, Abruzzi region (central Italy), in January 2016. PED virus (PEDV) identification was determined by real‐time RT‐PCR performed on RNAs purified from fecal samples collected from two symptomatic pigs. Whole genome sequence (PEDV 1842/2016) was also obtained by next generation sequencing straight from RNA purified from one fecal sample. Genome comparison with extant global PEDV strains revealed a high nucleotide identity with recently reported European and American S‐INDEL PEDVs. Efficient sequencing, share of genomic data combined with the implementation of epidemiological tools would be the ideal approach for study and analysis of transboundary infectious diseases as PED.

## Abstract 2

A porcine epidemic diarrhoea (PED) outbreak occurred in a swine fattening farm in Teramo province, Abruzzi region, in January 2016.Whole genome of the occurring PEDV was obtained straight from RNA purified from infected faeces. Genome comparison with extant global PEDV strains revealed a high nucleotide identity with recently reported European and American S‐INDEL PEDVs.

## Introduction

Porcine epidemic diarrhoea (PED) is an economically important and highly contagious enteric disease of swine, particularly in new born sucking piglets, in which strains of PED virus (PEDV) cause mortality up to 100% of infected individuals (Song *et al*. 2015).

PEDV is an enveloped, single‐stranded, positive sense RNA virus belonging to the family *Coronaviridae*, genus *Alphacoronavirus* (de Groot *et al*., 2011). Among viral proteins, the S, a glycoprotein, is responsible for viral attachment, entry and virulence (Sato *et al*. 2011). This protein is translated as a large polypeptide that is subsequently cleaved by virus‐encoded or host‐encoded proteases to produce two functional subunits, S1 and S2 (Ziebuhr *et al*. 2000). As for PEDV, the S1 domain is important for recognizing the porcine aminopeptidase N (pAPN), cellular receptor for PEDV, expressed on the surface of epithelial cells of small intestine (Li *et al*. 2007; Nam & Lee 2010). Moreover, the S1 domain was demonstrated to be a hypervariable region among PEDV strains as it is also for other coronaviruses (Decaro *et al*. 2009). The faecal‐oral route is the main route of PEDV transmission. Materials, equipment and feed contaminated with faeces and/or vomitus are also considered as major sources of infectious virus (Dee *et al*. 2014; Lowe *et al*. 2014). PEDV was first documented in the United Kingdom in 1971 (Oldham 1972) and then it spread to other European countries (prototype strain PEDV CV777). Between 2010 and 2013 severe outbreaks of PED were reported in China; they were caused by an emergent highly virulent PEDV strain (Li *et al*. 2012). In April 2013, virulent PEDV was also reported in US swine (Stevenson *et al*. 2013) causing severe epidemics across the whole country; it rapidly spread to Canada, Mexico, Caribbean and South America. These PED outbreaks induced significant economic losses for the US pork industry, causing the death of more than 8 million newborn piglets during a single year of epidemic period (Stevenson *et al*. 2013; Lee 2015). Indeed, nearly 10% of the nation's hog population was lost, severely reducing the supply of pork and sending bacon and pork chop prices to new records. Genetic analyses revealed high nucleotide (nt) identity between emerging United States and Chinese PEDV strains reported from 2010 to 2013, suggesting that US strains emerged because of spillover from China (Huang *et al*. 2013). Likely, PEDV had been introduced to United States from China by flexible intermediate bulk containers (Scott *et al*. 2016). In January 2014, a less virulent novel PEDV strain (prototype PEDV OH851) was detected in Ohio, USA (Wang *et al*. 2014). This new strain was mainly characterized by the presence in the hypervariable S1 protein coding sequence (CDS) of insertions and deletions. Accordingly, these new PEDV strains were called S‐INDEL PEDV.

In May 2014, PED was reported in a German fattening farm (Hanke *et al*. 2014). Thereafter, novel outbreaks were reported in other European countries including France (Grasland *et al*. 2015), Belgium (Theuns *et al*. 2015), The Netherlands (Van der Wolf *et al*. 2015), Italy (Efsa Ahaw Panel, 2014), Austria (Steinrigl *et al*., 2016), Spain (Carvajal 2015), Ukraine (Dastjerdi *et al*. 2015), Portugal (Mesquita *et al*. 2015) and Slovenia (Toplak *et al*. 2016). These outbreaks have all been sustained, with the remarkable exception of the Ukrainian strain, PEDV Poltava01/2014, by PEDVs related to the US S‐INDEL OH851 strain. In Italy, PED has been documented since 1997, with a few cases appearing per year. However, between 2005 and 2006, a severe PED epidemic occurred in Italy (Martelli *et al*. 2008), which was characterized by mortality rates in neonatal piglets of up to 34%, whereas it was very low in adults. During 2007–2012, only sporadic confirmed clinical cases of PED were reported, whereas at the beginning of 2015 a new severe epidemic of PED occurred (Boniotti *et al*. 2016). The disease mainly involved swine farms located in the north of Italy (Enrico Giacomini, IZSLER, personal communication). In this case report, we describe the detection and molecular characterization of a PEDV strain originated from a swine fattening farm in the Abruzzi region, Central Italy.

## Materials and Methods

In January 2016, a farm housing 1442 fattening pigs in the Teramo province of the Abruzzi region (central Italy) reported acute diarrhoea in pigs of all age groups. The farm is composed by five independent barns, 10 km distant from one another, though they share workers, materials, equipment and feed. In one of the barns (barn 1), it is located a pen for gestation, farrowing and nursery, in addition to other five pens doomed to the growth and fattening of weaned piglets. The other barns (barn 2‐5) have only growing‐finishing pens. Pens are usually divided into an outdoor area with partially slatted floor and an indoor area equipped with concrete solid floor. According to the attending veterinary physician, approximately the 50% of the animals developed clinical signs. The affected pigs showed anorexia, depression of the sensorium, profuse yellow‐greenish watery diarrhoea and varying degrees of weight loss and dehydration. Although the application of sanitary measures, within 1 week after the onset of the first clinical signs, the disease had spread to all age groups of pigs in all barns of the farm, but with major effects in piglets. Organic acids and electrolytes were administered to the animals in water. Very low mortality rates were observed. Indeed only 30 deaths occurred after 10 days since the initial onset of the disease, mainly in piglets and younger individuals. Clinical signs decreased within 1 week in the older pigs, but they persisted among the youngest individuals for approximately 4 weeks.

Two faecal samples were sent to the laboratories of the IZSAM for official diagnosis on January 26th. A faecal suspension was prepared for each sample using 1 g of faeces in a total volume of 10 mL of phosphate‐buffered saline solution with antibiotics; 100 *μ*L of the supernatants was used for RNA purification using BioSprint 96 One‐For‐All Vet Kit (Qiagen), following manufacturer's instructions. RNAs were tested by real‐time RT‐PCR (RT‐qPCR) using a commercial kit, ViroReal^®^ Kit PEDV & TGEV (Ingenetix). Whole‐genome sequencing was performed by combination of sequence independent single primer amplification method (SISPA) and next‐generation sequencing (NGS) following guidelines previously described from our group straight from RNA purified from one of the infected faecal sample (Marcacci *et al*. 2016). Deep sequencing was performed on the NextSeq 500 using the NextSeq 500/550 Mid Output Reagent Cartridge v2 (Illumina Inc.), 300 cycles and standard 150 bp paired‐end reads. To determine the genetic relationships with extant global PEDV strains, representative full‐length genome sequences of geographically and pathogenically distinct PEDV strains were retrieved from GenBank and aligned using MAFFT with default settings (Katoh *et al*. 2009). A phylogenetic tree was inferred using MEGA 6 software through the neighbor‐joining (NJ) distance method (TN93 model) performing bootstrap analysis with 1000 replicates (Tamura *et al*. 2013). Other tree‐building methods, including maximum parsimony and maximum likelihood, were used to verify the topology of the NJ tree. To include all extant partial S protein CDS of Italian PEDV strains, an additional NJ was inferred through the same model and bootstrap analysis including a total of 27 sequences of 539 bp including the S1 hypervariable region of the S protein CDS.

## Results

**Figure 1:**
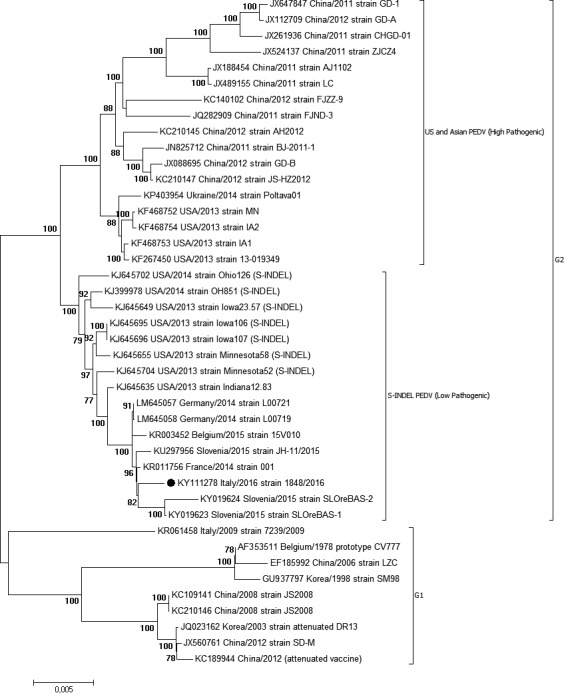
Neighbor‐joining phylogenetic tree based on full‐length genome alignment of 41 PEDV sequences downloaded from GenBank. Numbers along the branches indicate the percentage of 1000 bootstrap iterations. For each sequence, the strain designation, followed by country and year of isolation and the GenBank accession number is shown. PEDV sequence obtained in this study is indicated with a black circle.

**Figure 2:**
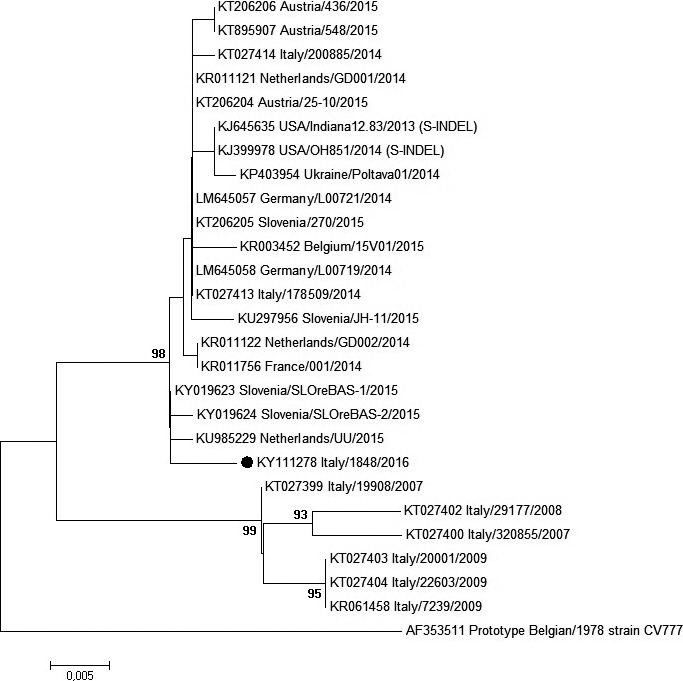
Neighbor‐joining tree inferred from multiple nt sequence alignment of the partial S1 domain (539 bp) of PEDV strains. Only bootstrap values ≥ 70, calculated on 1000 replicates, are indicated. Strain designation, country and year of detection and GenBank accession number are shown. PEDV sequence obtained in this study is indicated with a black circle.

Both samples were positive for PEDV RNA with a high load (C_*T*_‐value, 22) in faeces, while they tested negative for Transmissible Gastroenteritis Coronavirus and Porcine Deltacoronavirus. Whole‐genome sequence of strain 1842/2016 has been obtained and deposited in GenBank (accession number KY111278). By phylogenetic analysis of whole genomes, two distinct genogroups designated as genogroup 1 (G1) and genogroup 2 (G2) can be identified (Fig. 1). In G2, high pathogenic and low pathogenic strains (so called S‐INDEL PEDV) formed two separated groups as previously described (Vlasova *et al*. 2014; Lee 2015). PEDV 1842/2016 clustered in G2 and grouped together with the S‐INDEL PEDV strains recently reported in northern Europe (France 2014, KR11456; Germany 2014, LM645057‐LM645058; Belgium 2015, KR003452; Slovenia 2015, KY019623‐KU297956), with whom it shared nearly the 100% of nucleotide identity across the whole genome. Interestingly, the Ukrainian strain (Ukraine/Poltava01/2014, KP403954) did not cluster together with other European S‐INDEL PEDV but grouped with US and Asian high pathogenic strains, proving the presence in Europe of both high and low pathogenic PEDV strains. Moreover, the S protein of PEDV 1842/2016 showed three unique amino acid residues located at position 194, 206 and 674 with respect to extant publicly available PEDV sequences. As most of the Italian PEDV sequences available in GenBank are represented only by sequences of the hypervariable S1 region, we performed a further analysis, using a common sequence of 539 bp which includes this region. PEDV 1842/2016 showed 99% nucleotide identity with homologous regions of PEDV strains that have been circulating in Italy and Europe between 2014 and 2015 and 96–97% of nucleotide identity with Italian PEDV strains that circulated before 2014. Indeed, in the S1 tree (Fig. 2), currently circulating PEDVs (2014–2016) grouped together and separated from the “old” (2007–2009) Italian and European strains, which are, on the other hand, more closely related to the prototype European strain CV777, isolated in the 70s. Remaining genes are highly conserved among PEDV 1842/2016 and global S‐INDEL PEDV strains, sharing nearly the 100% of nucleotide identity (data not shown).

## Discussion

PED is not notifiable to the European Union (EU) or an OIE listed disease but it is now notifiable at the national level in several EU countries. It is progressively becoming a problem for the European swine industry. Thus, a fast generation and processing of data is required for efficient control and eradication activities. Next‐generation sequencing (NGS) is characterized by extremely parallel, cost efficient sequencing of genomic fragments, generating millions of short reads in a single sequencing run. NGS has opened new possibilities in the study of pathogen evolution, allowing researchers to track genomic changes over time. In this study, we characterized a PED outbreak which occurred in the Abruzzi region, Central‐Italy, in January 2016. The involved strain, PEDV 1842/2016, was demonstrated to be nearly identical to S‐INDEL PEDV strains which have been circulating in the last 2 years in Italy and in northern Europe. We strongly believe that prompt and efficient sequencing, share of genomic data combined with the implementation of epidemiological tools, with relevant genome characterization would be the ideal approach for study and analysis of transboundary infectious diseases as PED, even though they are not notifiable. In this way, we may have the possibility of assessing the risk associated to holdings based, for instance, on their trade of animals or feed, to support the veterinary services in tracing back and forward the animals in case of outbreaks of infectious diseases. Therefore, further efforts need to be spent at the European level for the implementation of novel holistic epidemiological tools. Considering the circulation of PEDV Ukraine/Poltava01/2014‐like virulent strains in the eastern part of Europe or the existence of virulent PEDV strains in USA and China, precise and accurate swine trade information from these areas is essential to assess proper countermeasures and mitigate the impact of highly virulent PEDV strains in naïve pigs.

## Source of funding

Funding was provided by the Italian Ministry of Health (Ricerca Corrente 2014 “Approccio metagenomico per una diagnosi rapida ed accurata di alcune infezioni batteriche e virali”). Mention of trade names or commercial products in this article is solely for the purpose of providing specific information and does not imply recommendation or endorsement by the IZSAM.

## Conflict of Interest

The authors declare that they have no competing interests.

## Ethical statement

The authors confirm their adherence to Veterinary Medicine and Science's Ethics Policy. However, ethical approval process and approval number were not required for the aims of the study.

## Contribution

Study design: MM, FC, GZ, NDA, AL; Laboratory and bioinformatic analyses: FP, MM, FC, AC, MF, MS, GZ, MO; Manuscript Draft: FP, FC, GZ, MM, AL.
